# Suppression of Filament Overgrowth in Conductive Bridge Random Access Memory by Ta_2_O_5_/TaO_x_ Bi-Layer Structure

**DOI:** 10.1186/s11671-019-2942-x

**Published:** 2019-03-28

**Authors:** Jie Yu, Xiaoxin Xu, Tiancheng Gong, Qing Luo, Danian Dong, Peng Yuan, Lu Tai, Jiahao Yin, Xi Zhu, Xiulong Wu, Hangbing Lv, Ming Liu

**Affiliations:** 1 Key Laboratory of Microelectronics Devices and Integrated Technology, Institute of Microelectronics, Chinese Academy of Sciences, Beijing, China; 2 School of Electronics and Information Engineering, Anhui University, Hefei, Anhui China

**Keywords:** Conductive bridge resistive switching memory (CBRAM), CMOS-compatible process, Bi-layer structure, Reliability

## Abstract

Bi-layer structure has been widely adopted to improve the reliability of the conductive bridge random access memory (CBRAM). In this work, we proposed a convenient and economical solution to achieve a Ta_2_O_5_/TaO_x_ bi-layer structure by using a low-temperature annealing process. The addition of a TaO_x_ layer acted as an external resistance suppressing the overflow current during set programming, thus achieving the self-compliance switching. As a result, the distributions of high-resistance states and low-resistance states are improved due to the suppression of the overset phenomenon. In addition, the LRS retention of the CBRAM is obviously enhanced due to the recovery of defects in the switching film. This work provides a simple and economical method to improve the reliability of CBRAM.

## Introduction

Conductive bridge resistive switching memory (CBRAM) is a breakthrough technology and is considered as next-generation non-volatile memory (NVM) due to its high scalability, simple structure, ease of 3D integration, and high-speed operation [1–3]. For practical application, the reliability issues, including the data retention and endurance, hinder the definitive introduction of these memory devices into the memory market. Structure engineering is the most popular approach to improve the reliability of CBRAM [4–7]. Zhao et al. confined cation injection to enhance CBRAM performance by nano-pore graphene layer [8]. Although the reliability of the device has highly improved, it makes costs of difficulty on material control and cannot be used in a standard CMOS process. In order to address this problem, Gong et al. proposed a CMOS-compatible and self-aligned method to form a CuSiN interfacial layer in Cu electrode for improving the low-resistance state (LRS) retention [9]. Cao et al. proposed a TiN barrier layer to improve the device reliability in CBRAM devices by eliminating the nano-filament overgrowth phenomenon and negative-SET behavior [10]. The above methods utilized the bi-layer structure to optimize the reliability of CBRAM effectively. However, they make costs of complex process flow or programming speed.

In this work, we propose a CMOS-compatible method to form a bi-layer device by a simple low-temperature annealing process. The double-layer device of Ta_2_O_5_/TaO_x_ structure was formed spontaneously, which shows better reliability characteristics compared with the un-annealed device. The enhanced reliability of the annealed device can be explained by the concentrated filaments formed along the grain boundary during programming. Furthermore, for a bi-layer annealing device, due to the existence of TaO_x_, the self-compliance behavior is achieved because the TaO_x_ layer serves as a resistor in series with a Ta_2_O_5_-resistive layer. This result provides a simple CMOS-compatible method to form a double-layer device and improve the reliability of CBRAM.

## Methods

The W plug with a diameter of 1 μm after CMP is served as the bottom electrode (BE). After depositing 5 nm Ta layer by DC magnetron sputtering, the Ta_2_O_5_ was formed through a thermal oxidation process, under 350 °C, in plasma O_2_ for 300 s by plasma-enhanced chemical vapor deposition (PECVD). Then, 40 nm Cu top electrode (TE) is sputtered and patterned by lithography. The memory cells are patterned through the etching process with a mixed gas of SF_6_ and C_3_F_8_ by using the TE as the hard mask. Afterward, the BE is extracted out by the Al pad. Finally, the device is completed with a CMOS-compatible low-temperature annealing process under 400 °C for 30 min. The size of the device is defined by the area of the bottom electrode, which is 1 μm^2^. As a reference, the device without the annealing process is also prepared. The electrical DC measurements are carried out by using a Keithley 4200-SCS semiconductor parameter analyzer. For all measurements, the voltage is applied to the Cu TE with the W BE grounded.

## Results and Discussion

**Fig. 1:**
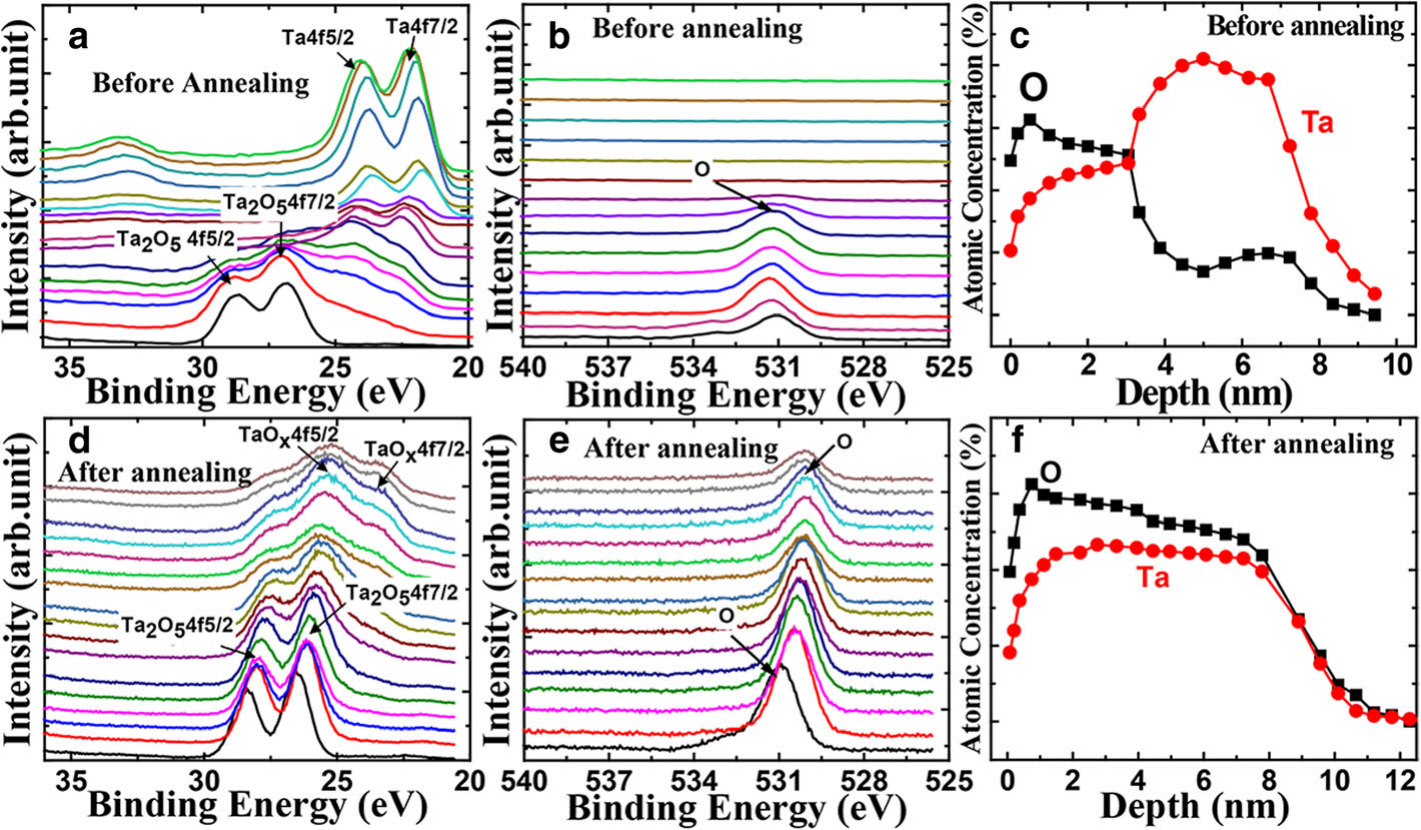
The XPS shows depth profile of Ta before (**a**) and after (**d**) annealing. **b**, **e** Depth profile of O before and after annealing, respectively. **c, f** Atomic concentration profile of O and Ta with depth before and after annealing, respectively

For a profound insight into the annealing process, the composition and chemical bonding state in the Ta_2_O_5_ films before and after annealing process are analyzed by X-ray photoelectron spectroscopy (XPS). The etch rate of the sample is 0.5 nm/point. In Fig. 1a, the peaks of Ta_2_O_5_ 4f doublet with peak binding energies of 26.70 eV (Ta_2_O_5_ 4f_7/2_) and 28.60 eV (Ta_2_O_5_ 4f_5/2_) with peak separation of 1.9 eV are observed at the surface [11–13]. This case demonstrates the existence of Ta_2_O_5_ layer.

With the depth increasing, the peaks of Ta_2_O_5_ 4f doublet disappear and the peaks at 22.33 eV, 23.96 eV corresponding to Ta 4f_7/2_, Ta 4f_5/2_ appear. Figure 1b verifies that there is no O signal at the same depth where the Ta 4f_7/2_ and Ta 4f_5/2_ exist. In other words, there is metallic Ta on the surface of Ta_2_O_5_ for the un-annealed device. The depths of the Ta_2_O_5_ and Ta analyzed from Fig. 1c are 4 nm and 2.5 nm, respectively. In addition, there is the peak of the O atomic concentration in the depth of 7 nm, indicating the existence of the absorbed oxygen. Figure 1d and e show the depth profiles of XPS spectra from the Ta_2_O_5_ films after the annealing process. The peaks of Ta 4f doublet and Ta_2_O_5_ 4f doublet exist together at a certain depth. The intensity of the Ta^5+^ oxidation state gradually weakens with the increasing depth. Combined with the all-around oxygen signal along the film depth, we confirm that the TaO_x_ exists on the surface of Ta_2_O_5_ [11, 14]. Calculated from Fig. 1f, the thickness of the Ta_2_O_5_ is 4 nm and TaO_x_ is 3.5 nm. Therefore, the TaO_x_ is formed by changing the adsorbed oxygen to lattice oxygen in the annealing process. The oxygen re-distribution would reach a saturation point saturated after the annealing process. The thickness of TaOx as well as the Forming voltage will not increase even though the annealing time increases, proving the large process margin of this annealing process.

**Fig. 2:**
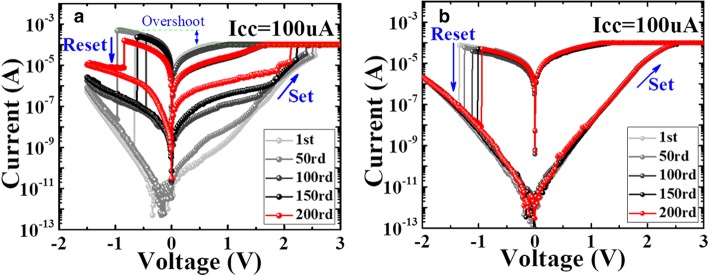
Typical I-V curves of Cu/TaOx/W devices before annealing (**a**) and after annealing (**b**) with 200 cycles

**Fig. 3:**
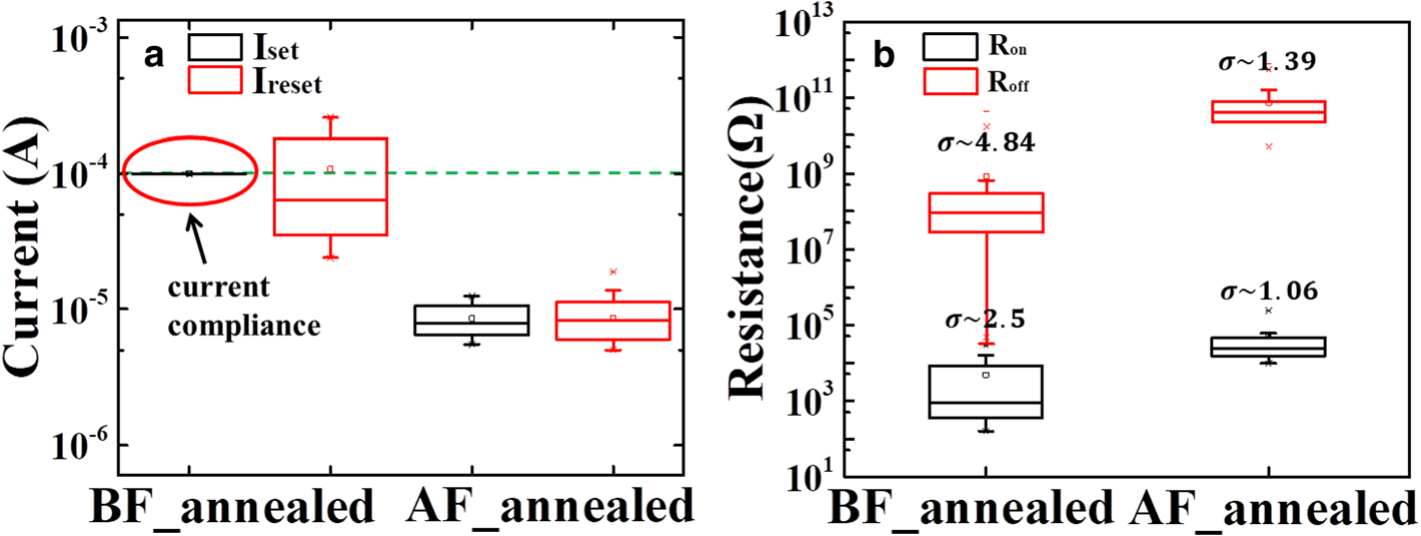
**a** Set and RESET Current distributions before and after annealing, respectively. **b** The resistance distribution of HRS and LRS before/after annealing

Figure 2a and b are the resistive switching characteristics of Cu/Ta_2_O_5_/W before and after annealing under DC sweeping mode. The initial resistances (*R*_initial_) of the two devices are both in high-resistance state (HRS) with values of ~ 10^9^ Ω and 10^10^ Ω, respectively. The higher *R*_initial_ of the annealed device is due to the thicker oxide film formed under thermal process. Notably, this device does not need a forming process, which is quite expected in practical application. For the un-annealed device, it switches to LRS abruptly when the applied voltage reached to a critical value during positive voltage sweeping. Some ultra-low LRS occurred during the set process. The RESET current in such case is much higher than the pre-set compliance current, indicating the overshoot phenomenon happened in this device. Figure 3b exhibits the unstable LRS and HRS within 200 cycles for the un-annealed device. The large variation between cycle-to-cycle leads to the memory window reduced to be as small as 20. Figure 2b shows the switching behavior of the annealed devices. The current flowing through the cell increases gradually and reaches the compliance current. No obvious switching point is observed, avoiding the overshoot phenomenon happened in the un-annealed devices. A memory window as high as 10^4^ was achieved during the switching cycles, owing to the uniform distribution of HRS and LRS.

The suppression of the overset phenomenon in the annealed device could also be verified by the improved distribution of the RESET current (*I*_RESET_) and Set current (*I*_Set_) in the annealed device, as shown in the Fig. 3a. The *I*_Set_ of the un-annealed device is stuck at the *I*_CC_ but *I*_RESET_ distributes widely. In contrast, for the annealed device, the *I*_RESET_ is similar to *I*_Set_. The device-to-device uniformity is evaluated by analyzing the *R*_on_ and *R*_off_ in 20 different devices under DC mode. As shown in Fig. 3 (b), the *R*_on_ extracted under *V*_read_ of 0.1 V for the un-annealed device distributes from 10^2^ Ω to 10^5^ Ω, while the *R*_on_ of the annealed device distributes from 10^4^ Ω to 10^5^ Ω. The relatively higher *R*_on_ of the annealed device resulted from the series resistance of the TaO_x_ layer. Moreover, the HRS distribution of the annealed device is also much improved. As shown in Fig. 3b, the standard deviation (SD) of *R*_off_ is reduced from 4.84 to 1.39.

**Fig. 4:**
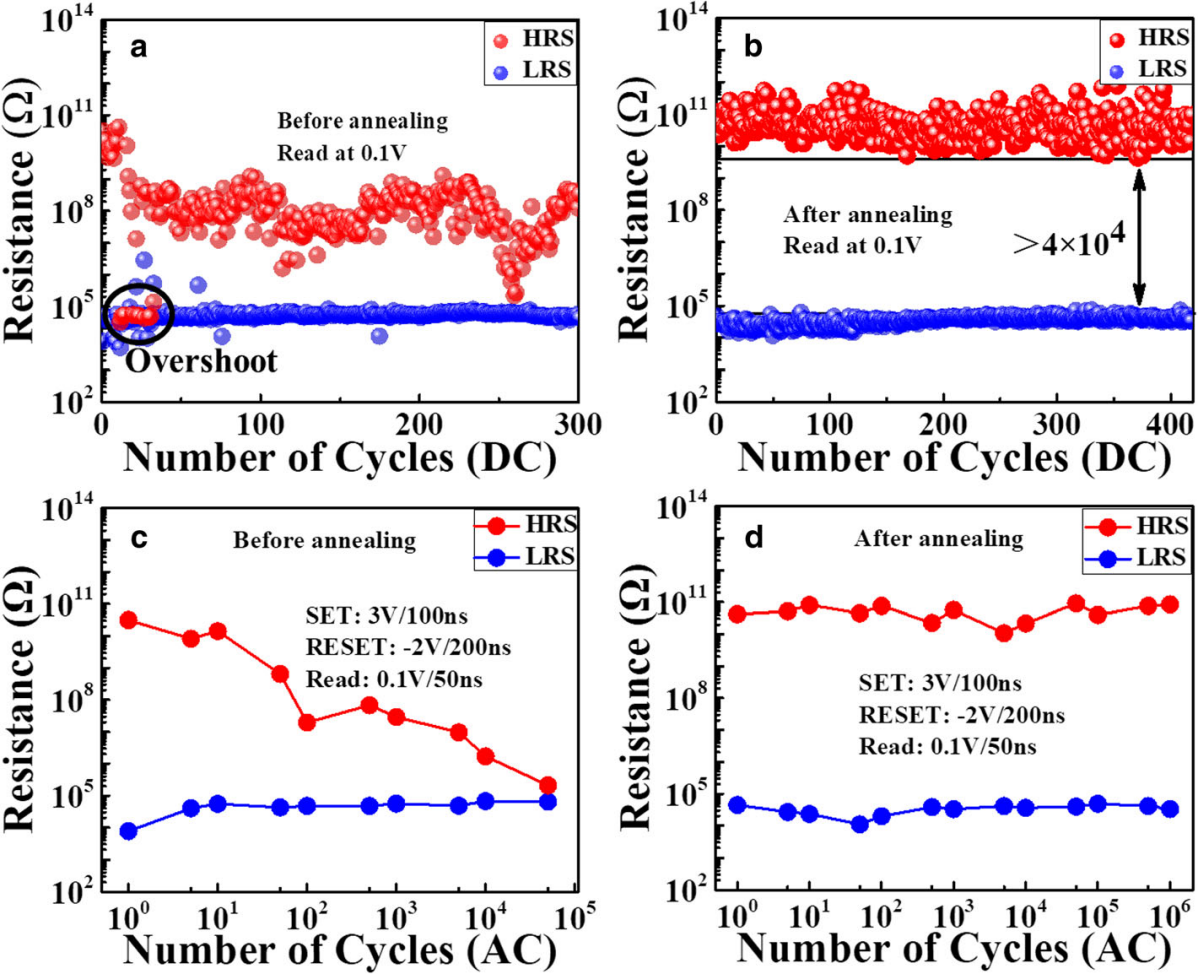
The cycling results of **a** the devices without annealing under 300 DC cycles and **b** the devices with annealing under 400 DC cycles. **c, d** Endurance characteristics under AC mode with the optimized operation configuration: set 3 V/100 ns; RESET − 2 V/200 ns. Up to 10^6^ cycles were obtained for the device after annealing

The cycling results under DC sweeping are shown in Fig. 4a and b. For the un-annealed device, the HRS/LRS ratio is around 10^5^ at first, and then decreases gradually and finally sticks at LRS. Note that a few soft errors could be observed during cycling, in the form of HRS (red dots) and LRS (blue dots) run back and forth occasionally. For the annealed device, the HRS/LRS ratio remains stable (~ 10^4^) without any degradation. During pulse measurements, the proper pulse programming conditions are optimized as 3 V/100 ns for set operation, − 2 V/200 ns for RESET operation, and 0.1 V/50 ns for read operation. The sensing time for Set/RESET/Read operation is 15 ns/12 ns/25 ns, respectively. As can be seen from Fig. 4c, the endurance for the un-annealed device is usually less than 5 × 10^4^ switching cycles. However, from Fig. 4d, it is surprising that the annealed device still works well without failure after more than 10^6^ switching cycles. Based on our previous study [15], the endurance failure in CBRAM is related to the unstable RESET operation resulted from the filament overgrowth into the counter electrode. On the one hand, the overgrown filament needs more energy to rupture and tends to cause incomplete RESET and lower HRS. On the other hand, the overgrowth of filament into the counter electrode leads to residual Cu ions in the counter electrode, which could serve as a reservoir of metal ions and make unexpected negative-SET. For the annealed device, the filament overgrowth is well suppressed by the incorporation of TaO_x_ layer and results in more stable RESET operation. As a result, the memory window is well maintained and the cycling characteristic is much improved.

**Fig. 5:**
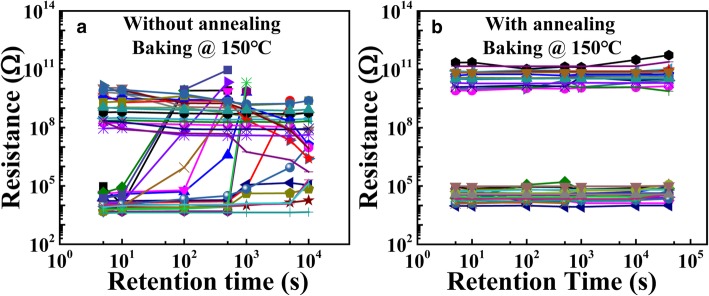
Retention characteristics of the HRS/LRS for **a** un-annealed device and **b** annealed device at 150 °C

Considering the retention characteristic plays a crucial role for practical application of CBRAM [16]. The retention characteristics are measured under 150 °C using the vacuum oven. The resistance of each cell is checked after cooling down to room temperature at every decade interval. Figure 5a and b show the dependence of the R_HRS_/R_LRS_ on the baking time for the device without annealing and with annealing, respectively. For the un-annealed devices (Fig. 5a), as the time increases, the devices failed gradually within 10^4^s. However, for the annealed device (Fig. 5b), among the recorded 20 devices, the resistances of the LRS and HRS do not show any degradation as the baking time increases. That is to say, the retention of the devices is highly improved by the annealing process. The lifetime of the annealed device at 85 °C could be extracted as 10 years by Arrhenius plot, which is in good accordance to the CBRAMs reported [17, 18]. The achievement of better retention characteristic for the annealed device is because the annealing process recovers some defects in the switching film, which would slow down the diffusion of the Cu species.

**Fig. 6:**
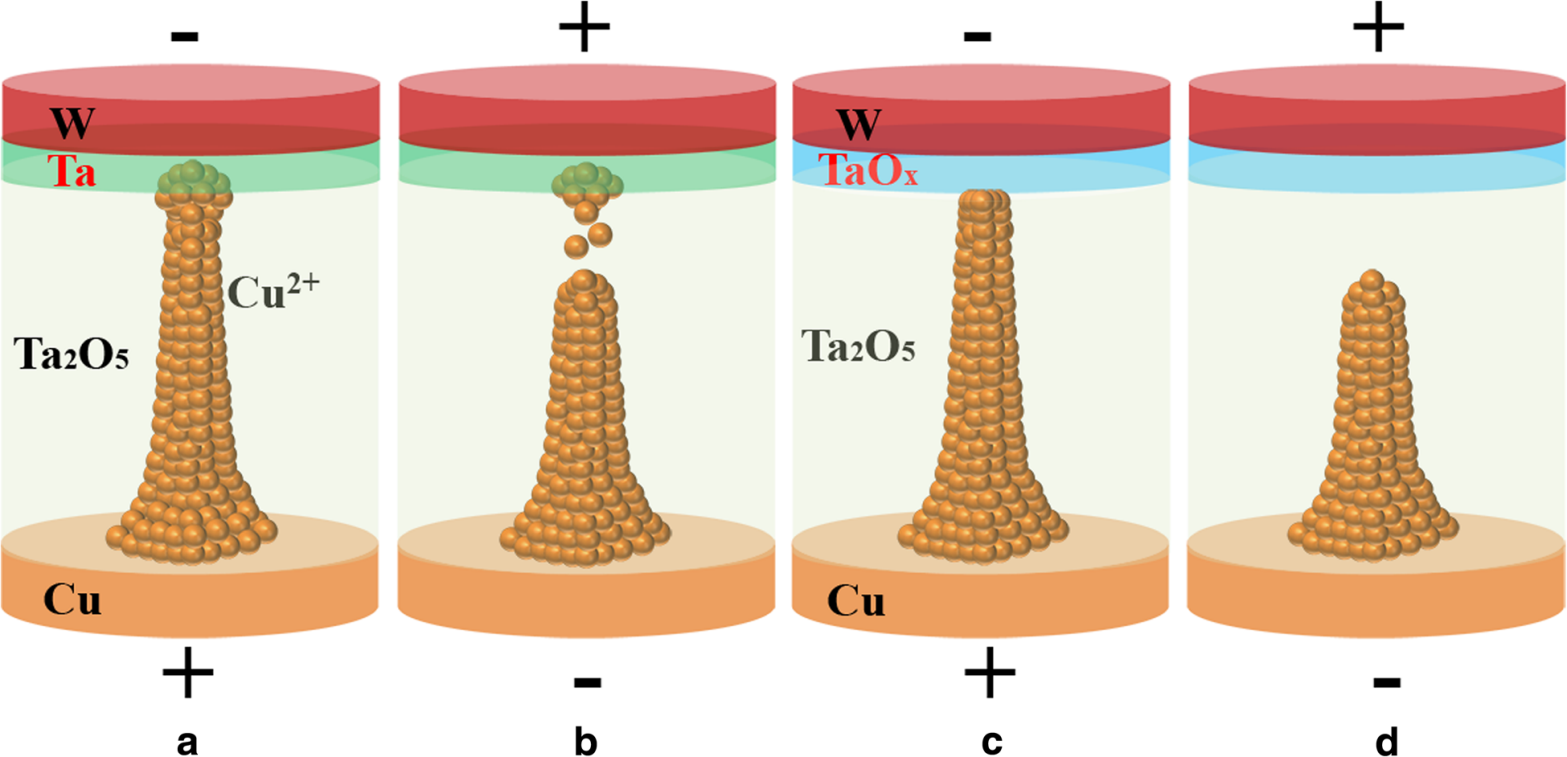
The physical modeling for the switching behavior of the annealed and un-annealed devices. The **a** Set and **b** RESET process for the un-annealed device with the structure of Cu/Ta_2_O_5_/Ta/W. **c** Set and **d** RESET process for the annealed device with the structure of Cu/Ta_2_O_5_/TaO_x_/W. The filament overgrowth is suppressed by the TaO_x_ layer formed during the annealing process

Based on the above results, a physical model for the switching behavior of the annealed and un-annealed devices is illustrated in Fig. 6a–d. The filament growth in CBRAM is associated with the Cu ion transportation in the lattice of electrolyte [19]. The overshoot phenomenon that happened in the un-annealed device makes filament overgrowth into the counter electrode. During the RESET operation, the residual Cu ions stored in the counter electrode will drift into the tunnel gap between the filament tip and the counter electrode, resulting in the residual Cu^+^ at the end of the RESET operation and serious variation of HRS. As the diffusion coefficient of Cu in TaO_x_ (4.9 × 10^− 20^ cm^2^/s) is much less than that in Ta (1.0 × 10^− 6^ cm^2^/s), the Cu diffuses into TaO_x_ is much more difficult under the electric field during Set operation in the sample of Cu/Ta_2_O_5_/TaO_x_/W [20, 21]. Hence, the overset behavior and filament overgrowth could be well suppressed, and the RESET operation becomes more stable.

## Conclusions

In this letter, we investigated the switching characteristics of a TaO_x_-based CBRAM device. A Ta_2_O_5_/TaO_x_ bi-layer stack was formed after a post thermal annealing treatment. The TaO_x_ layer could act as an external resistance suppressing the overflow current during set operation. Both HRS and LRS distribution are greatly improved due to the suppression of the overset phenomenon. Moreover, the data retention of the CBRAM is enhanced due to the recovery of defects in the switching film during thermal annealing. This work provides the most convenient and economical solution to achieve the bi-layer structure and improve the reliability of CBRAM.

## Abbreviations

CBRAM: Conductive bridge random access memory
HRS: High-resistance states
LRS: Low-resistance states
NVM: Non-volatile memory
PECVD: Plasma-enhanced chemical vapor deposition
TE: Top electrode
